# Bias in Estimation of a Mixture of Normal Distributions

**DOI:** 10.4172/2155-6180.1000179

**Published:** 2013-11-23

**Authors:** Spencer Lourens, Ying Zhang, Jeffrey D Long, Jane S Paulsen

**Affiliations:** 1 Department of Biostatistics, University of Iowa, Iowa City, IA, 52442, USA; 2 Departments of Psychiatry and Biostatistics, University of Iowa, Iowa City, IA, 52442, USA; 3 Departments of Psychiatry, Neurology and Psychology, University of Iowa, Iowa City, IA, 52442, USA

**Keywords:** Biomarkers, Disparity index, EM algorithm, Mixtures

## Abstract

Estimating parameters in a mixture of normal distributions dates back to the 19th century when Pearson originally considered data of crabs from the Bay of Naples. Since then, many real world applications of mixtures have led to various proposed methods for studying similar problems. Among them, maximum likelihood estimation (MLE) and the continuous empirical characteristic function (CECF) methods have drawn the most attention. However, the performance of these competing estimation methods has not been thoroughly studied in the literature and conclusions have not been consistent in published research. In this article, we review this classical problem with a focus on estimation bias. An extensive simulation study is conducted to compare the estimation bias between the MLE and CECF methods over a wide range of disparity values. We use the overlapping coefficient (OVL) to measure the amount of disparity, and provide a practical guideline for estimation quality in mixtures of normal distributions. Application to an ongoing multi-site Huntington disease study is illustrated for ascertaining cognitive biomarkers of disease progression.

## Introduction

Finite mixture (or mixing) distributions refer to composite distributions constructed by mixing a number (K) of component distributions. Estimation of mixture distributions is a classical statistical problem which has been studied for over 100 years. The first account of mixture data being analyzed was documented by Pearson [1] in 1894. Pearson analyzed data of 1000 crabs, consisting of ratios of forehead to body length, from the Bay of Naples by deriving a series of equations using Methods of Moments in order to estimate parameters denoting crab characteristics in the case where the number of components is two (K=2). The word mixture is used because the density function of a random observation is a mixing of several (distinct) component density functions of the form $f(x)=\sum_{i=1}^{K}\eta_{i}f_{i}(x)$, with $\sum_{i=1}^{K}\eta_{i}=1$ and *η_i_*>0 for all i=1, 2, …, K, and *f_i_*(*x*), i=1, …, K being distinct densities with known form but each contains one or more unknown parameters. Each observation comes from one of the K (distinct) component distributions with unknown membership status. The aim of mixture modeling is to estimate the parameters of each component density, *f_i_*, as well as the mixing parameters, *η_i_*. This problem can also be regarded as a missing data problem, as the group membership or the component distribution from which an observation is generated is not known. We would not only like to be able to estimate the parameters from a mixture, but also to gain an understanding of estimation performance over a wide range of settings. Mixtures have been used to analyze data arising in Hydrology [2], Economics [3], Ecology [1], Bioinformatics [4], as well as many other fields [5]. Partial mixtures, for which group membership is known for specific subjects in the data set, have also been considered [6].

Several estimation methods for normal mixtures have been proposed in the literature. Among them, the ordinary maximum likelihood estimation method (MLE) appears to be a straightforward choice, as the likelihood for the mixture is easy to establish. However, directly calculating the MLE via optimizing the likelihood for a mixture of normal distributions is difficult and numerical algorithms can lead to computational issues such as non-convergence, as noted in Xu and Knight [3]. Hosmer [6] developed an estimation method for the case K=2, which can be viewed as a special case of the well-known Expectation-Maximization (EM) algorithm for computing the MLE in missing data problems [7]. He found that the MLE may not perform well with regards to bias in the small sample case, especially when the two distributions are poorly separated. Leytham [2] corroborated Hosmer's work in regards to the estimation of the means and variances in normal mixtures through simulation, but claimed that estimation of quantiles for normal mixtures may be approximately unbiased. Moreover, results regarding the MLE for normal mixtures are inconsistent in the literature. Some researchers report unbiased estimation via the MLE [8], but others conclude otherwise [2,3]. Mixtures of normal distributions may have a model identifiability issue when K is unknown a priori and the likelihood can be unbounded for some special cases [9,10]. Moreover the EM algorithm may be converging to a local maximum of the likelihood and may then yield biased estimation for model parameters of interest. In response to the unbounded likelihood of normal mixtures, alternative methods such as the Moment Generating Function (MGF) method [11], the Discrete Empirical Characteristic Function method (DECF) [12], and the Continuous Empirical Characteristic Function method (CECF) [3] have been proposed. They can be viewed as special cases of Generalized Methods of Moments (GMM) [10]. Limited simulation studies have provided some numerical evidence for the merit of these methods compared to MLE [3]. The question is whether this set of GMM methods performs better than the MLE in general.

Our study of mixture distributions is motivated by empirical analysis with progression marker data in Huntington Disease (HD). HD is an autosomal dominant neurodegenerative disease caused by the trinucleotide cytosine-adenine-guanine (CAG) expansion in the gene of the protein huntingtin. Clinical symptoms of HD include progressive motor dysfunction, cognitive decline, and psychiatric disturbance [13]. Individuals who have a CAG repeat of length 36 or greater are referred to as at-risk of HD, and individuals who are at-risk of HD but have not yet received the HD motor diagnosis are described as being prodromal-HD (prHD). As the disease progresses, prHD individuals exhibit impairments noted above, often with daily functioning deterioration as a result, and patients with larger CAG repeats often deteriorate at much faster rates than those with smaller CAG repeats. Although the CAG expansion is vital for determining the at-risk status of an individual, it is estimated that fewer than 5% of those at-risk of having a CAG expansion length of 36 or greater (i.e., having at least one parent diagnosed with HD in their lifetime) are willing to undergo the genetic testing to ascertain their at-risk status. It is reported that those who do seek testing do so in order to determine how to make future choices regarding family lives and careers. Among the risks of a positive gene test are suicides, genetic discrimination, and stress or other psychological disturbance [14]. As a result, at-risk status information is not always known, and in the past, researchers have used proxies for at-risk status. For example, Langbehn et al. [15] used the information that at least one parent was diagnosed with HD as a proxy for at-risk status. However, using such information as a proxy can result in biased estimation and invalid inference for understanding disease progression in HD. We believe that discovering critical HD progression biomarkers to serve as a proxy for the at-risk status of HD for individuals who are unwilling to undergo gene testing is important, because these individuals can receive assistance in dealing with progression, such as counseling and therapy to avoid the negative impacts mentioned above (suicide, genetic discrimination, etc.) associated with a positive result from the gene test for HD, and also because of the surprisingly low (5%) prevalence of at-risk individuals being willing to undergo genetic testing. The task is to study the distributions of potential HD progression biomarkers for both HD at-risk (prHD) and healthy control cohorts when the information of CAG is unknown, either because of stigmas associated with a positive gene test, or because the information was too expensive for collection.

In this article, we review the existing methods for estimating normal mixtures and conduct an overarching numerical experiment to examine their estimation performance with focus on comparing the bias between the MLE via EM algorithm and CECF method. In addition to the numerical experiment, we apply the methods to HD data from the PREDICT-HD study. Intuitively, the estimation of a mixture distribution should be largely influenced by the disparity between the individual component distributions. We use the overlapping coefficient (OVL) [16] to define a disparity index for quantifying the difference between the two component distributions, and we study the estimation performance over a wider range of this disparity index than considered by previous authors. We aim to provide a practical guide for the validity of the methods in terms of estimation bias in relation to the disparity index.

The rest of the paper is organized as follows. Section 2 provides an overview of the competing methods proposed in the literature and provides a disparity index to quantify the difference between two distributions. Section 3 presents an extensive simulation study comparing the performance of the MLE via the EM algorithm and the CECF method under various settings, along with the index values. Section 4 applies this index to PREDICT-HD data to ascertain HD cognitive biomarkers as potential proxy variables for HD at-risk status. Section 5 gives our concluding remarks and some guidance regarding analyzing normal mixtures.

## Overview of the Methods for Estimation of a Normal Mixture

In this section, we provide an overview of the estimation methods mentioned in Section 1, specifically in the case that the data come from a mixture of two component normal distributions. Suppose we observe a random sample of continuous outcomes *Y*_1_, *Y*_2_, …,*Y*_n_ that are distributed according to a normal mixture of $N(\mu_{1},\sigma_{1}^{2})$ and $N(\mu_{1},\sigma_{2}^{2})$. Let **R_1_** and **R_2_** denote the two latent groups, D*_i_*=1[*Y_i_* ∈ **R**_2_] the indicator for outcome Y*_i_* coming from Group 2 for *i*=1, 2, …, *n*, and *η*=*P*[*Y_i_* ∈ **R_2_**], the probability that *Y_i_* comes from Group 2. In the mixture problem, the information of group membership *D_1_*, *D_2_*, …, *D_n_* is unknown and *η*, the mixing parameter, must also be regarded as unknown in the analysis. For the two component normal mixture, the probability density function (PDF) for *Y_i_*, *i*=1, 2, …,*n* is:

(1) $$f_{\text{yi}}(y)=\eta f_{2}(y)+(1-\eta )f_{1}(y)i=1,2,\ldots ,n$$

where

$$f_{j}(y)=\frac{1}{\sqrt{2\pi \sigma_{j}}}\exp\left(-\frac{{\left(y-\mu_{j}\right)}^{2}}{2\sigma_{j}^{2}}\right)\;j=1,2,$$

with $\sigma_{1}^{2}$ and $\sigma_{2}^{2}$ bounded below by a small constant ξ > 0.

## MLE via the EM algorithm

Although the likelihood for the unknown parameter $\theta =(\eta ,\mu_{2},\mu_{1},\sigma_{2}^{2},\sigma_{1}^{2})$ can be easily established for the observed data with the PDF given in (1), the numerical algorithm for computing the MLE is not stable, as demonstrated in Xu and Knight [3]. In a mixture setting, we do not observe the complete data, (*Y*_i_, *D*_i_), for each subject, as component membership (*D*_i_) is missing for all individuals under study. This means that mixture problems can be considered missing data problems. Since this is a missing data problem, the EM algorithm is a natural alternative for computing the MLE. To apply the EM algorithm, the “complete” data likelihood is formed as if *D_i_* are observed. It turns out the log complete likelihood is a linear function of unobserved data *D_i_* for *i*=1, 2, …,*n*. Hence, the conditional expectation of each latent observation *D*_i_, given the observed data and current estimate of unknown parameters, needs to be evaluated and then be substituted into the (log) complete likelihood for *D*_i_. The EM algorithm is particularly effective for this situation, because both the E-step and the M-step have explicit solutions as given by Leytham [2] and Nityasuddhi and Bohning [8]. We briefly present their solutions here. Given a current estimate of $\theta ,{\hat{\theta }}_{\left(k\right)}=({\hat{\eta }}_{\left(k\right)},{\hat{\mu }}_{2,(k)},{\hat{\mu }}_{1,(k)},{\hat{\sigma }}_{2,(k)}^{2},{\hat{\sigma }}_{1,(k)}^{2})$, the estimate θ̂_(*k*+1)_ can be explicitly updated by

$$\begin{array}{l} {\hat{\eta }}_{\left(k+1\right)}=\frac{\sum_{i=1}^{n}q_{\text{ik}}}{n}\\ {\hat{\mu }}_{2,(k+1)}=\frac{\sum_{i=1}^{n}q_{\text{ik}}y_{i}}{\sum_{i=1}^{n}q_{\text{ik}}}\\ {\hat{\mu }}_{1,(k+1)}=\frac{\sum_{i=1}^{n}(1-q_{\text{ik}})y_{i}}{\sum_{i=1}^{n}\left(1-q_{\text{ik}}\right)}\\ {\hat{\sigma }}_{2,(k+1)}^{2}=\frac{\sum_{i=1}^{n}q_{\text{ik}}{\left(y_{i}-{\hat{\mu }}_{2,(k)}\right)}^{2}}{\sum_{i=1}^{n}q_{\text{ik}}}\\ {\hat{\sigma }}_{1,(k+1)}^{2}=\frac{\sum_{i=1}^{n}(1-q_{\text{ik}}){\left(y_{i}-{\hat{\mu }}_{1,(k)}\right)}^{2}}{\sum_{i=1}^{n}\left(1-q_{\text{ik}}\right)} \end{array}$$

where

$$q_{\text{ik}}=E(D_{i}|y_{i};{\hat{\theta }}_{\left(k\right)})=\frac{{\hat{\eta }}_{\left(k\right)}{\hat{f}}_{2,(k)}(y_{i})}{{\hat{\eta }}_{\left(k\right)}{\hat{f}}_{2,(k)}(y_{i})+(1-{\hat{\eta }}_{\left(k\right)}){\hat{f}}_{1,(k)}(y_{i})}$$

with ${\hat{f}}_{j,(k)}(y)=\frac{1}{\sqrt{2\pi {\hat{\sigma }}_{j,(k)}}}\exp\left(-\frac{{\left(y-{\hat{\mu }}_{j,(k)}\right)}^{2}}{2{\hat{\sigma }}_{j,(k)}^{2}}\right)$ for j=1,2. Randomly choosing an initial value, θ̂_(0)_, procedure can be easily implemented and forced to stop when the L_2_-distance of the full vector of parameters in adjacent iterations is sufficiently small, say less than 10^-6^.

## The CECF method

Motivated by the estimation method involving minimizing a distance between the empirical characteristic function and the population-based characteristic function originally proposed by Heathcote [17], Xu and Knight [3] developed the CECF method. For observed continuous outcomes **y**=(*y*_1_, *y*_2_, …,*y*_n_), they consider minimizing

(2) $$c(\theta ,y)=\int_{-\infty }^{+\infty }{\left\|C_{n}(y,r)-C(\theta ,r)\right\|}^{2}G(r)\text{dr}$$

where $C_{n}(y,r)=\sum_{j=1}^{n}\exp({\text{iry}}_{i})/n$ denotes the empirical characteristic function, C (*θ*, *r*)=*E*(exp(*ir*Y)) the characteristic function, and G(*r*) a weight function. Specifically, for a normal mixture,

$$C(\theta ,r)=\eta \exp(i\mu_{2}r-\sigma_{2}^{2}r^{2}/2)+(1-\eta )\exp(i\mu_{1}r-\sigma_{1}^{2}r^{2}/2).$$

Therefore, the choice of G(*r*)=exp(-*br*^2^) makes (2) integrable and results in an explicit function of unknown parameter *θ* and the tuning parameter *b*. Heathcote [17] did not consider the optimal choice of *b*, and instead set it to 1, as was commonly done in the past for this type of problem. For a given value *b*, the minimization problem (2) is straightforward. Xu and Knight [3] chose the optimal *b* by iteratively solving for the *θ* value that minimizes (2) at a given *b* and updating the value *b* at the value which minimizes the trace (or determinant) of the resulting variance matrix for the current estimate of *θ*. This procedure continues until the change in the optimal *θ* values is sufficiently small. They demonstrated, in a limited simulation study, that the CECF method is comparable to the standard MLE in terms of estimation efficiency. They also showed that when the two component distributions have the same mean, the MLE procedure leads to numerical nonconvergence but the CECF is still a numerically valid method. In fact, we believe the MLE procedure was probably not implemented effectively in Xu and Knight [3]. Our numerical experiment showed that the MLE method works well for Cases D_1_ and D_2_ considered in Xu and Knight [3], if the EM algorithm is adopted to compute the MLE.

## The DECF method

Similar to the CECF method, the DECF method considers minimizing the distance between sample quantities and population analogs over a fixed set of grid points, *r*=(*r*_1_, *r*_2_ …, *r_m_*). That is, the unknown parameter θ is estimated by minimizing

(3) $$e(\theta ,y,r)=\sum_{i=1}^{m}{\left\|C_{n}(y,r_{i})-C(\theta ,r_{i})\right\|}^{2}$$

where C_n_(y, *r_i_*) and C (θ, *r_i_*) are the same as defined for the CECF method. The performance of the DECF methods depends on the choice of grid points ***r***, both the number and location of the nodes, *r_i_*, *i*=1, …, *m*. Work has been done to show that as the grid becomes finer and more extended, the DECF becomes more efficient [10] and the DECF method is actually a special case of GMM. Multiple authors [10,12] have noted that the estimation efficiency of the DECF could be increased by rescaling the weight matrix used by GMM (the weight matrix is the identity matrix in the presentation above). The CECF method is generally preferred over the DECF, as the distance is defined over the whole continuum of ***r*** values in (−∞, ∞) and hence, it does not require specification of the grid points ***r***.

## The MGF method

The MGF method developed in Quandt and Ramsey [11] is very similar in nature to the DECF method. The only difference is that the moment generating function is used to replace the characteristic function of the DECF method. That is, the unknown parameter θ is estimated by minimizing

(4) $$m(\theta ,y,r)=\sum_{i=1}^{m}{\left(M_{n}(y,r_{i})-M_{n}(\theta ,r_{i})\right)}^{2}$$

Where $M_{n}(y,r)=\sum_{i=1}^{n}\exp({\text{ry}}_{i})/n$ and $M(\theta ,r)=\eta \exp(\mu_{2}r+\sigma_{2}^{2}r^{2}/2)+(1-\eta )\exp(\mu_{1}r+\sigma_{1}^{2}r^{2}/2)$. Again choice of the number of points and their location must be made to facilitate the use of this method. Moreover, the possible non-existence of the moment generating function for fat-tailed distributions (i.e., Cauchy) makes the MGF method less desirable than the DECF method in practice [12].

## A disparity index

For a mixture distribution, estimation quality largely depends on the difference between the component distributions. If the distributions have a large overlap it will be difficult to identify the group membership of observations and to estimate each component's parameters. Therefore, it is highly desirable to define an index which measures the difference between the two latent distributions in order to develop a guideline regarding estimation quality for a mixture distribution.

For normal mixtures, Hosmer [6] defined an index,

$$H=\frac{\left\|\mu_{2}-\mu_{1}\right\|}{\min(\sigma_{2},\sigma_{1})}$$

to measure the separation between the two normal distributions. This measure, however, cannot capture divergence of the two latent normal distributions due to a difference in variance alone. The simulation study conducted in Hosmer [6] only considered the performance of MLE for the case of *μ*_1_ ≠ *μ*_2_. Whenever *μ*_1_=*μ*_2_, *H* ≡ 0 regardless of the variances. For the case of σ_2_>σ_1_, with σ_2_ increasing, it will be shown via simulation that estimation quality improves, eventually resulting in negligible bias. However, the value of *H* will not change in this situation, thus, *H* does not properly index the observed improvement in estimation performance. As a result of these observations, the proper term for describing the difference between two normal distributions that make up the mixture distribution is “disparity”. The disparity between two distributions not only accounts for mean separation, but also for differences in variability. One measure that considers both the means and the variances is Nityasuddhi's *D* [8], which is defined as,

$$D=\frac{1}{2}\left[\sum_{i=1}^{2}{\left(\mu_{i}-\bar{\mu }\right)}^{2}+\sum_{i=1}^{2}{\left(\sigma_{i}^{2}-{\bar{\sigma }}^{2}\right)}^{2}\right]$$

where *μ̄* = (*μ*_1_ + *μ*_2_)/2 and ${\bar{\sigma }}^{2}=(\sigma_{1}^{2}+\sigma_{2}^{2})/2$. However, this index can yield similar values for two opposing cases in which estimation quality will be very different. For instance, similar *D* values may result due to a difference in means, while the variances are the same, or due to a difference in variances, while the means are the same. That is to say, the same *D* value may be observed when only the variances differ, or when only the means differ. Our simulation shows that much smaller differences in means are necessary for estimation to have negligible bias, while differences in variances must be larger for estimation to have negligible bias. Thus, two different underlying parameter values may yield the same Nityasuddhi's *D* value, even if estimation performance varies substantially in both cases.

Ideally, a good disparity index should always have a large value when estimation quality is good, and a small value when estimation is bad. Intuitively, the shared (or overlapping) area under the two normal distributions is key to determining the estimation quality, as the observations from this area obscure their group membership.

Distributions with little overlap tend to be easily separated and result in parameter estimation with small bias. However, for mixtures where the component distributions have large overlap, severe bias might result. Inman and Bradley [16] have studied the *OVL* for the case of normal distributions and derived an explicit formula to calculate its value,

(5) $$\text{OVL}=\left\{ \begin{array}{ll} 2\Phi (-\frac{\left|\mu_{1}-\mu_{2}\right|}{\sigma }) & \text{if}\sigma_{1}=\sigma_{2}=\sigma \\ 1+\Phi \left(\frac{\tau_{1}-\mu_{1}}{\sigma_{1}}\right)+\Phi \left(\frac{\tau_{2}-\mu_{2}}{\sigma_{2}}\right)-\Phi \left(\frac{\tau_{1}-\mu_{2}}{\sigma_{2}}\right)-\Phi \left(\frac{\tau_{2}-\mu_{1}}{\sigma_{1}}\right) & \text{if}\sigma_{1}\neq \sigma_{2} \end{array}\right.$$

In (5), Φ denotes the cumulative distribution function of the standard normal distribution, τ_1_ and τ_2_ are given by

$$\begin{array}{cc} & \tau_{1}=\frac{\mu_{1}\sigma_{2}^{2}-\mu_{2}\sigma_{1}^{2}-\sigma_{1}\sigma_{2}{\left[{\left(\mu_{1}-\mu_{2}\right)}^{2}+(\sigma_{2}^{2}-\sigma_{1}^{2})\log(\frac{\sigma_{2}^{2}}{\sigma_{1}^{2}})\right]}^{1/2}}{\sigma_{2}^{2}-\sigma_{1}^{2}}\\ \text{and} & \tau_{2}=\frac{\mu_{1}\sigma_{2}^{2}-\mu_{2}\sigma_{1}^{2}+\sigma_{1}\sigma_{2}{\left[{\left(\mu_{1}-\mu_{2}\right)}^{2}+(\sigma_{2}^{2}-\sigma_{1}^{2})\log(\frac{\sigma_{2}^{2}}{\sigma_{1}^{2}})\right]}^{1/2}}{\sigma_{2}^{2}-\sigma_{1}^{2}} \end{array}$$

**Figure 1:**
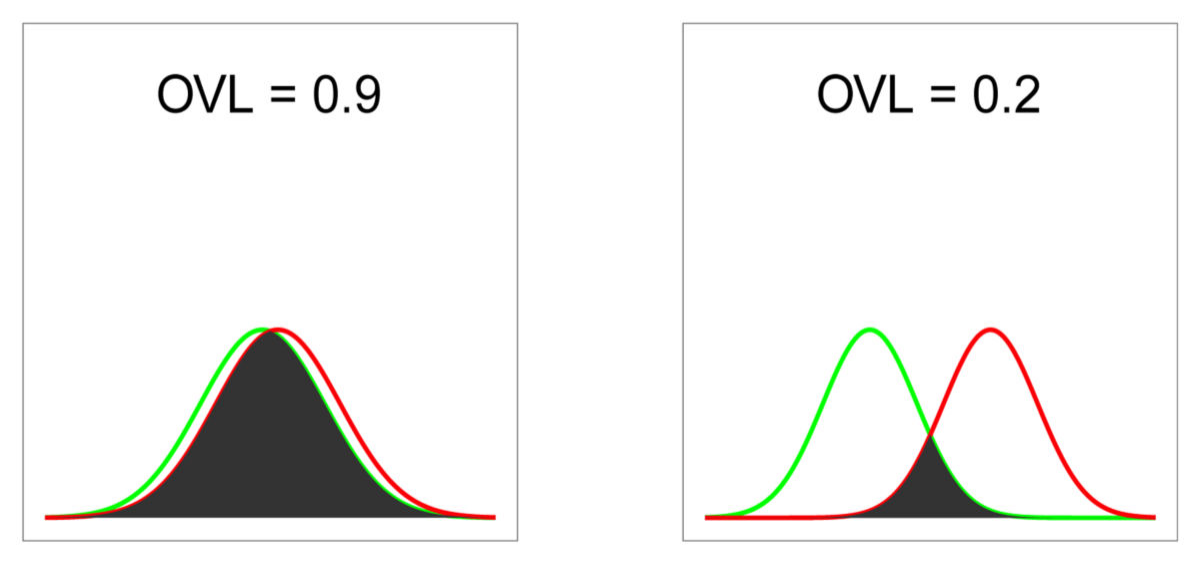
OVL Example Plots-the shaded portion depicts the overlap: the left panel corresponds to a small disparity of DI=0.1; the right panel to a large disparity of DI=0.8.

We propose the use of *DI*=1−*OVL* as a disparity index. Note that *DI* satisfies our requirements specified in the above paragraph. Namely, large values of *DI* (large disparity) reflect cases where estimation quality is good, and small values of *DI* (small disparity) reflect cases where estimation quality is poor. This index does not suffer from the sub-optimal properties of the indices mentioned above, as it allows for variances alone to contribute to the disparity between the two normal distributions. Two examples of normal mixture distributions, one with large disparity (*DI*=0.8) and one with small disparity (*DI*=0.1), are shown in Figure 1.

## Simulation Study

**Figure 2:**
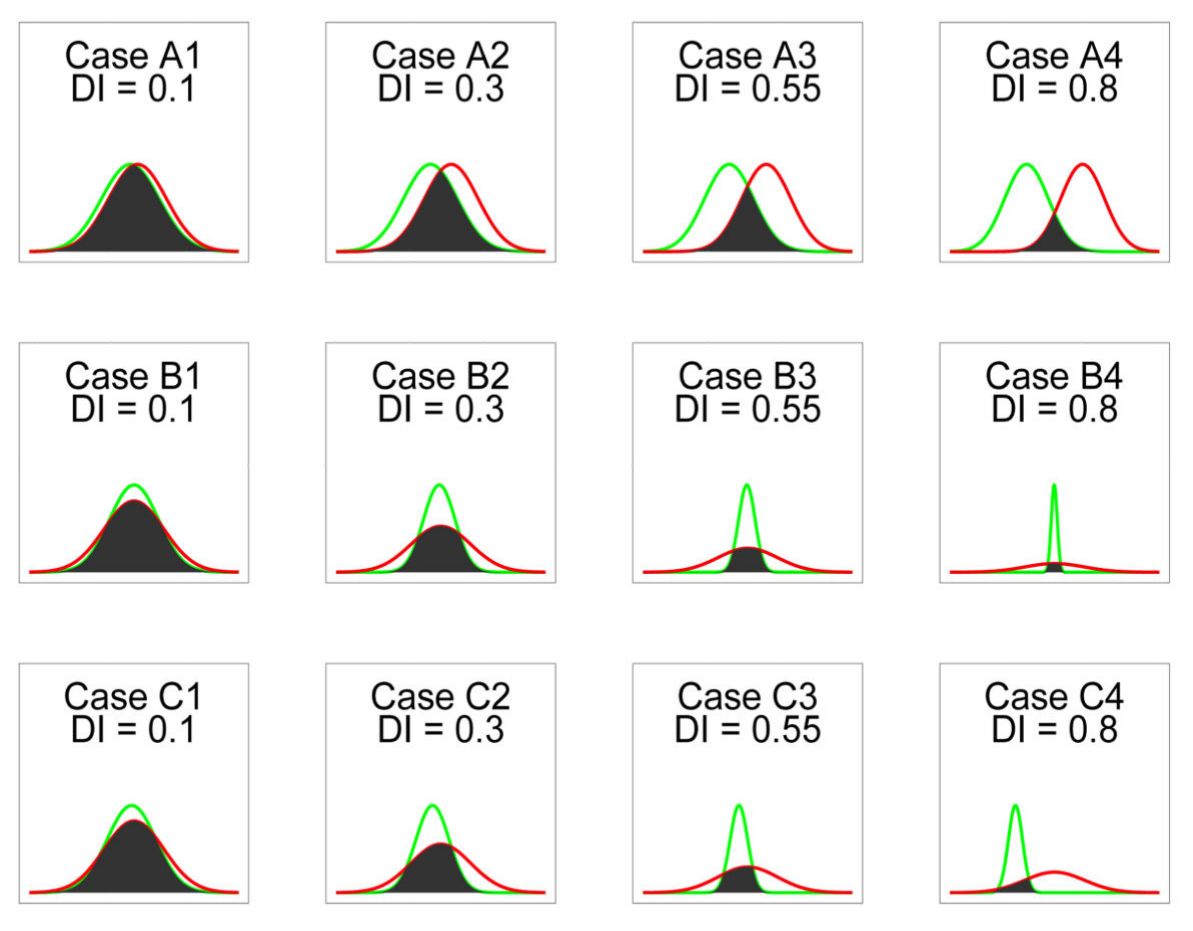
Overlaps of the two distributions under the simulation settings-the graphs are not on the same numerical scale.

We conduct a comprehensive simulation study to examine the estimation performance of the methods discussed above with focus on the estimation bias. As the CECF, DECF, and MGF methods are very similar in nature and the CECF has the merit of not requiring the identification of the optimal number and locations of the grid points, we only include the CECF in the study and compare it to the MLE (via the EM algorithm). *DI* is used to quantify the amount of disparity between the two component distributions. For simulations, the model parameters are given in Table 1 and they characterize the amount of disparity due to mean and variance differences in various scenarios. Figure 2 provides a visual display of the two component distributions for the cases given in Table 1 with the shading depicting distribution overlap. It is worth noting that our study covers a much broader range of disparity values than any other studies conducted in the literature [3,6,8].

For each case listed in Table 1, we conducted a Monte Carlo simulation study with 1000 trials. The estimation bias and Monte Carlo Standard Deviation (MCSD) were calculated based on the results from the 1000 trials and reported in Tables 2-4. Because the estimation bias and standard deviation for the variance parameters are very sensitive to the actual values, relative bias and relative MCSD for the variance parameters are reported in the tables. Relative bias is defined as the bias divided by the value of the parameter being estimated. Relative MCSD is defined in an analogous manner. For example, if the bias and MCSD are 0.05 and 1.50, respectively, and the parameter value is 2, then the relative bias is 0.025 and relative MCSD is 0.75. It is worth noting that for approximately 5% of the trials for cases with the least disparity, the EM algorithm for the MLE did not lead to numerical convergence, while the CECF method did not have any numerical problems. When this occurred, data were regenerated and the simulation continued until 1000 trials were completed. This convergence issue was not observed in cases with at least moderate disparity between the two component distributions.

Table 2 summarizes the simulation results for Case A with sample sizes 100, 200 and 500. It appears that there is substantial bias in estimating both mean and variance parameters using the MLE when *DI* is less than or equal to 0.55. When *DI* ≤ 0.55 CECF has smaller bias in estimating the means but larger bias in estimating the variances compared to the MLE. Particularly, for the case of *DI*=0.55, the estimation of μ_2_ for MLE and the estimation of $\sigma_{1}^{2}$ for CECF are still noticeably biased even when *n*=500. When there is a large amount of disparity between the two component distributions, for instance when *DI*=0.8 in our study, both MLE and CECF work very well. However, the MLE method outperforms the CECF method with regards to both estimation bias and standard error. This is not surprising, as the MLE is the efficient estimation method when it works.

Table 3 summarizes the simulation results for Case B with sample sizes 100, 200 and 500, respectively. In this scenario, the bias in estimation of the means is small and for the most part negligible for all cases for the MLE, but it is not the case for the CECF when *DI*=0.1. Clearly when *DI*=0.1, the MLE outperforms the CECF, but both methods are too biased in estimating the variances to be useful in practice. When *DI*=0.3, the estimation bias appears to be acceptable for the MLE if sample size is greater than or equal to 200. But the estimation bias for the smaller variance is still relatively large for the CECF. When *DI* ≥ 0.55, the estimation bias is virtually negligible for all the model parameters under both methods, but the MLE is apparently preferred over the CECF as it has smaller MCSD. The estimation bias of the mixing parameter η is relatively small for the MLE, even in the case of small disparity. As the disparity increases, the MLE works better than the CECF, in terms of having smaller MCSD. However, estimation of the mixing parameter is highly variable (under both methods) in cases with small disparity (*DI* ≤ 0.3).

Table 4 summarizes the simulation results for Case C with sample sizes 100, 200 and 500, respectively. When both means and variances are allowed to vary between the two component distributions, the simulation results are similar to Case A. That is, in the small sample or small disparity cases (*n*=100, 200 or *DI*=0.1, 0.3), the CECF tends to have smaller estimation bias for the means but larger bias for variances. When the amount of disparity is large (*DI* ≥ 0.55), the MLE is clearly the winner between the two competing methods.

As a concluding remark for the simulation study, the MLE may be generally preferred over the CECF when a variance difference is the source of the disparity between the two distributions or when *DI* is large, say greater than or equal to 0.55. However, use of the MLE in cases where *DI* is less than 0.55 requires caution, particularly when the disparity between the distributions is purely due to a separation of the means. Though the CECF is an alternative method for estimating the means with less bias than the MLE when separation of the means is the source of small disparity between the distributions, it still results in biased estimation for the means when *DI* is small. In general, the MLE is a better method for estimating the variances than the CECF, as it results in less estimation bias as well as smaller standard error.

## Application to the PREDICT-HD Data

The PREDICT-HD study is an ongoing observational study of prHD participants at 32 sites in the United States, Canada, Australia, Germany, Spain and the United Kingdom [18]. Comprehensive longitudinal data have been collected, including more than 80 variables from over 1300 research participants who underwent genetic testing for the HD mutation. As mentioned in the introduction, if an individual's CAG repeat length is greater than or equal to 36, this individual is considered at-risk for HD and is referred to as prodromal-HD (prHD) if no HD diagnosis is received.

We apply the *DI* to the PREDICT-HD data with the aim of identifying possible sensitive cognitive biomarkers that may distinguish between prHD (case) individuals and healthy controls (control), particularly when CAG repeat length is masked or unknown. If CAG repeat length is not observed for individuals under study, then the observed data are a mixture from the control group and the case group. The size of the study sample and the longitudinal nature of the study may facilitate opportunities to discover disease biomarkers whose progress may indicate an individual's at-risk status without knowledge of their CAG repeat length. We focus on the following five cognitive measures: Symbol Digit Modalities Test (SDMT), Stroop Color Test (STROOP-C), Stroop Word Test (STROOP-W), Trail Making Test A (TRAILS-A), and Trail Making Test B (TRAILS-B).

SDMT [19] involves a simple substitution task to pair specific numbers with given geometric figures within a fixed amount of time. Individuals with cerebral dysfunction usually perform poorly on the SDMT, which is indicated by a smaller value of this measure. The task of the Stroop tests [20] is to look at pages of colored words, reading words or naming colors as quickly as possible within a fixed amount of time. A smaller value of these measures indexes the individual's speed of processing. The Trails A test [21], a measure of speeded attention, requires individuals to draw the lines connecting the numbers 1,2,3,4 etc. in order until reaching the end. The Trails B test [21] asks individuals to draw the lines connecting the numbers 1,2,3,4 etc. and the letters A,B,C,D etc. in alternating order. The total time (in seconds) needed to complete each of these tasks is recorded. A larger value is indicative of cognitive slowing and difficulty shifting cognitive sets.

The PREDICT-HD study has collected these cognitive measures longitudinally for both prHD individuals and healthy controls. Having at-risk information allows us to estimate the group characteristics (*μ*_2_, *μ*_1_, etc.) and their corresponding *DI* values empirically. This gives us a better idea of the amount of disparity between the two distributions that is indicative of estimation quality. For this analysis, denote **R_1_** and **R_2_** the two latent groups with **R_1_** representing the control group and **R_2_** the prHD group. The parameters of interest are: $\theta =(\eta ,\mu_{2},\mu_{1},\sigma_{1}^{2},\sigma_{2}^{2})$, and parameters with subscript 2 correspond to the prHD group, while those with subscript 1 correspond to the control group. Since PREDICT-HD records CAG length, we can estimate the means and variances using their sample estimates and substitute them into (5) to obtain an estimate of *OVL*, *OVL*^⋆^ and calculate the *DI* by *DI*^⋆^=1−*OVL*^⋆^. The results are summarized in Table 5.

**Figure 3:**
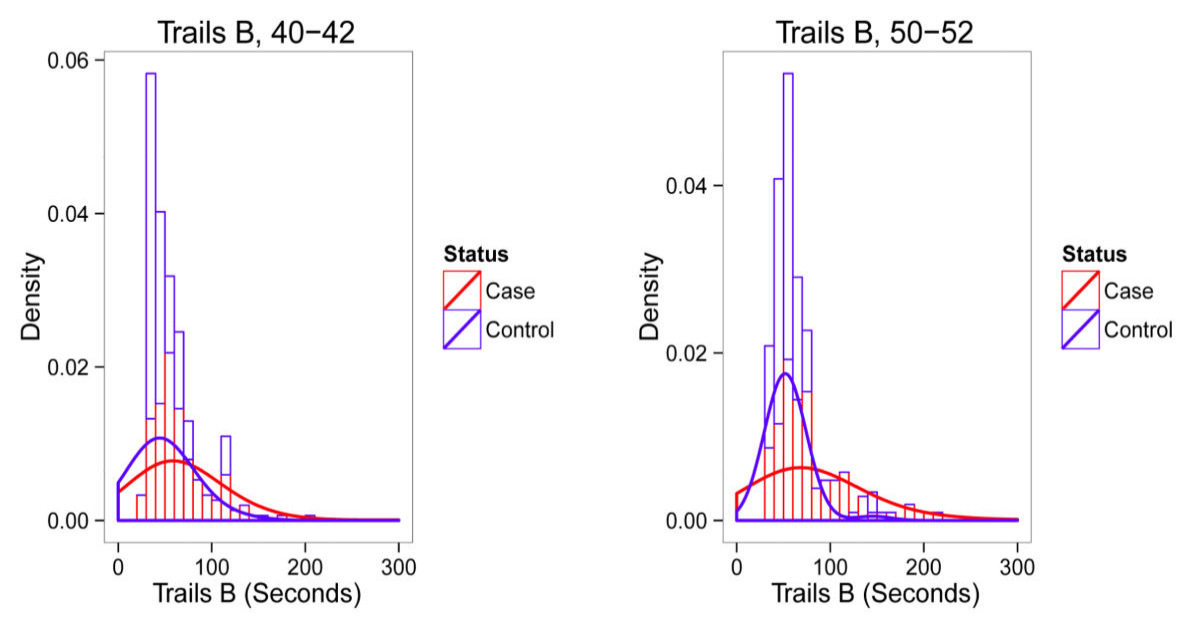
The distributions of Trails-B scores.

Table 5 presents the characteristics of the two cohorts and their corresponding *DI*^⋆^ for the five cognitive measures mentioned above at two age windows: 40-42 and 50-52. We considered the 40-42 age window and 50-52 age window so that we could determine whether the ability to estimate parameters for prHD and control individuals changed over time (average age of HD onset is approximately 40). We anticipated that there would be more disparity between the prHD and control groups by age 50-52, as prHD individuals will have had more time to progress, which would be reflected by higher *DI* values and thus, better estimation performance. Based on the simulation results presented above, it appears that only the Trails-B measure in the age window 50-52 may have a chance of providing a reasonable estimate of the model parameters when the genetic information of CAG repeat length is unknown or not considered, because the disparity for Trails-B in the 50-52 window is the largest (*DI*^⋆^=0.465). To estimate all parameters, including means/variances/the mixing proportion, we then apply both the MLE (via the EM algorithm) and the CECF methods to the Trails-B data, as if CAG repeat length were not observed, and compare their performance in light of our simulation results presented above. These estimates are summarized in Table 6. This real data example resembles the simulation scenario C3 with sample size around 100 where the estimation is less biased for the CECF method. In this setting, we can only compare our results with the empirical estimates given in Table 5, since we do not know the true parameter values. The empirical estimates serve as a reference for comparison as they are asymptotically efficient. Indeed, the CECF method yields closer estimates than the MLE method when inspecting the values given at the bottom row of Table 5. Nevertheless, the resulting estimates for the mixing parameter are far from the sample proportion $\hat{\eta }=\frac{104}{145}=0.717$ for both methods. This is mainly due to the fact that the estimation standard error is quite large for *η̂* and implies that when there is quite a bit of overlap between the two distributions, the methods may yield reasonable estimates for the mean and variance parameters but could have difficulty correctly estimating the mixing parameter. Both methods largely overestimate *µ*_2_, and slightly underestimate σ_1_. The histograms and density estimations for TRAILS-B data at the two age windows are plotted in Figure 3. It appears that the assumption of normal mixture is not unreasonable when ignoring some outliers in both groups and the two underlying distributions are not very separated. The poor estimation result given in Table 6 is anticipated based on the simulation results, as *DI* that is associated with the results shown in Table 6 is only 0.465 and the sample size is 145.

## Final Remarks

Estimation of normal mixtures is a classical problem that has been widely researched. While the MLE and the CECF appear to be the most popular methods, their estimation properties have not been extensively studied. This is probably due to the well-known fact that the normal mixture can be an ill-posed model when the disparity between the component distributions is small [1,21]. In this article, we utilize the OVL to quantify the disparity between the two distributions and then empirically examine when the methods can lead to reasonable estimates of the model parameters. The results provide an instructive guideline regarding the use of these methods. Generally speaking, when there is enough disparity, the MLE is still a more favorable method in practice, particularly when a difference in variances is the major source of the disparity between the two component distributions. When a difference in means is the major source of the disparity, the MLE may not lead to estimation with negligible bias if *DI* is small, and in this case, the CECF may be a reasonable alternative.

Our simulation study implies that neither the MLE nor the CECF method will yield a satisfactory outcome with regards to accurately estimating parameters for prHD individuals and healthy controls based on cross- sectional cognitive measures in PREDICT-HD data, as the *DI* values for these measures are too small at the times considered. The amount of overlap present led to the CECF and MLE largely overestimating the mean for prHD individuals and underestimating the amount of variability in the control group, relative to the empirical estimates when the at-risk status is known. Since HD is a progressive disease, investigating the disparity between longitudinal trajectories of these cognitive measures between the prHD and healthy control may provide a better indication on how well the model parameters can be estimated. A future research direction is to develop an index which measures the disparity between the two groups based on longitudinal data. Latent class modeling of generalized linear mixed-effects models could be used for the groups' longitudinal trajectories, in order to identify sensitive cognitive markers for indirectly ascertaining at-risk status in similar cohorts.

## Figures and Tables

**Table 1:** Simulation Settings-the model parameters used in the simulation study.

Case	*DI*	*η*	μ_2_	μ_1_	$\sigma_{2}^{2}$	$\sigma_{1}^{2}$
A1	0.1	0.5	1.25	1	1	1
A2	0.3	0.5	1.77	1	1	1
A3	0.55	0.5	2.51	1	1	1
A4	0.8	0.5	3.56	1	1	1
B1	0.1	0.5	1	1	1.5	1
B2	0.3	0.5	1	1	3.6	1
B3	0.55	0.5	1	1	13.2	1
B4	0.8	0.5	1	1	101	1
C1	0.1	0.5	1.1	1	1.48	1
C2	0.3	0.5	1.5	1	3.22	1
C3	0.55	0.5	2.2	1	11.5	1
C4	0.8	0.5	6.55	1	18.0	1

**Table 2:** Comparison of estimation bias and standard deviation between MLE and CECF-Case A.

		Bias (MCSD)									
		MLE method					CECF method				
Case	n	*η*	μ_2_	μ_1_	$\sigma_{2}^{2}$	$\sigma_{1}^{2}$	*η*	μ_2_	μ_1_	$\sigma_{2}^{2}$	$\sigma_{1}^{2}$
**A1**	**100**	0.013	0.521	-0.519	-0.374	-0.383	-0.031	0.461	-0.363	-0.398	-0.352
		(0.299)	(0.718)	(0.664)	(0.424)	(0.443)	(0.327)	(0.636)	(0.570)	(0.586)	(0.562)
**DI=0.1**	**200**	0.016	0.394	-0.436	-0.275	-0.301	-0.008	0.434	-0.312	-0.338	-0.357
		(0.283)	(0.644)	(0.614)	(0.414)	(0.412)	(0.341)	(0.558)	(0.510)	(0.549)	(0.510)
	**500**	0.060	0.254	-0.441	-0.143	-0.266	-0.007	0.325	-0.313	-0.321	-0.309
		(0.268)	(0.508)	(0.590)	(0.396)	(0.393)	(0.357)	(0.538)	(0.518)	(0.493)	(0.583)
**A2**	**100**	0.017	0.320	-0.343	-0.321	-0.327	-0.010	0.186	-0.183	-0.336	-0.301
		(0.359)	(0.759)	(0.699)	(0.476)	(0.493)	(0.336)	(0.660)	(0.668)	(0.628)	(0.988)
**DI=0.3**	**200**	0.032	0.228	-0.258	-0.212	-0.221	0.002	0.117	-0.134	-0.285	-0.261
		(0.301)	(0.727)	(0.644)	(0.443)	(0.524)	(0.349)	(0.621)	(0.594)	(0.587)	(0.698)
	**500**	0.055	0.131	-0.236	-0.106	-0.173	0.001	0.078	-0.091	-0.257	-0.255
		(0.280)	(0.701)	(0.608)	(0.413)	(0.448)	(0.367)	(0.540)	(0.569)	(0.519)	(0.510)
**A3**	**100**	-0.007	0.196	-0.117	-0.202	-0.156	-0.009	0.063	-0.040	-0.228	-0.169
		(0.293)	(0.776)	(0.689)	(0.508)	(0.538)	(0.316)	(0.698)	(0.679)	(0.635)	(0.709)
**DI=0.55**	**200**	-0.012	0.171	-0.079	-0.127	-0.077	0.005	0.013	-0.062	-0.146	-0.136
		(0.290)	(0.740)	(0.638)	(0.479)	(0.490)	(0.325)	(0.645)	(0.646)	(0.629)	(0.599)
	**500**	-0.025	0.131	-0.026	-0.088	-0.023	0.008	0.003	-0.035	-0.098	-0.100
		(0.278)	(0.605)	(0.557)	(0.407)	(0.414)	(0.301)	(0.552)	(0.560)	(0.493)	(0.474)
**A4**	**100**	-0.005	0.021	-0.007	-0.040	-0.024	-0.007	-0.014	0.038	-0.005	0.041
		(0.177)	(0.473)	(0.485)	(0.492)	(0.501)	(0.210)	(0.509)	(0.518)	(0.680)	(0.708)
**DI=0.8**	**200**	-0.005	0.021	0.005	-0.013	0.009	-0.011	0.014	0.028	-0.013	0.046
		(0.130)	(0.342)	(0.345)	(0.361)	(0.373)	(0.151)	(0.356)	(0.376)	(0.459)	(0.502)
	**500**	-0.001	0.004	0.003	-0.002	0.011	-0.007	0.011	0.019	-0.010	0.040
		(0.082)	(0.206)	(0.211)	(0.219)	(0.230)	(0.095)	(0.224)	(0.240)	(0.267)	(0.301)

**Table 3:** Comparison of estimation bias and standard deviation between MLE and CECF (0.001* denotes values less than 0.001)-Case B.

		Bias (MCSD)									
		MLE method					CECF method				
Case	n	*η*	μ_2_	μ_1_	$\sigma_{2}^{2}$	$\sigma_{1}^{2}$	*η*	μ_2_	μ_1_	$\sigma_{2}^{2}$	$\sigma_{1}^{2}$
**B1**	**100**	-0.038	0.005	-0.038	-0.287	-0.326	0.022	0.005	-0.471	0.055	-0.493
		(0.286)	(1.283)	(0.590)	(0.463)	(0.403)	(0.341)	(1.073)	(0.590)	(0.678)	(0.424)
**DI=0.1**	**200**	-0.011	-0.045	-0.035	-0.211	-0.288	0.061	0.030	-0.497	0.076	-0.488
		(0.290)	(1.199)	(0.567)	(0.459)	(0.379)	(0.356)	(0.973)	(0.569)	(0.527)	(0.433)
	**500**	-0.028	-0.070	-0.082	-0.132	-0.164	0.071	0.007	-0.490	0.097	-0.431
		(0.279)	(1.143)	(0.444)	(0.513)	(0.338)	(0.373)	(0.899)	(0.523)	(0.497)	(0.452)
**B2**	**100**	-0.035	-0.072	-0.001*	-0.135	-0.081	0.032	-0.009	0.017	-0.098	-0.283
		(0.276)	(1.595)	(0.414)	(0.514)	(0.589)	(0.315)	(1.287)	(0.467)	(0.686)	(0.634)
**DI=0.3**	**200**	-0.030	0.043	-0.004	-0.010	-0.024	0.062	0.019	-0.002	-0.016	-0.251
		(0.244)	(1.218)	(0.267)	(0.436)	(0.521)	(0.304)	(0.973)	(0.348)	(0.652)	(0.618)
	**500**	-0.001	-0.012	0.001*	0.033	-0.022	0.062	-0.010	0.001	0.011	-0.176
		(0.185)	(0.414)	(0.147)	(0.266)	(0.370)	(0.255)	(0.515)	(0.228)	(0.436)	(0.481)
**B3**	**100**	-0.007	-0.002	0.013	-0.001*	0.024	0.010	-0.017	0.019	-0.010	-0.066
		(0.137)	(0.865)	(0.227)	(0.313)	(0.619)	(0.146)	(0.922)	(0.229)	(0.382)	(0.560)
**DI=0.55**	**200**	-0.001	0.013	0.004	0.003	0.006	0.013	0.010	0.005	-0.012	-0.050
		(0.089)	(0.385)	(0.155)	(0.203)	(0.359)	(0.097)	(0.424)	(0.165)	(0.243)	(0.390)
	**500**	0.001	0.009	0.004	-0.007	0.004	0.006	0.003	0.004	-0.014	-0.020
		(0.055)	(0.243)	(0.095)	(0.123)	(0.195)	(0.060)	(0.269)	(0.099)	(0.141)	(0.225)
**B4**	**100**	-0.003	0.015	0.008	-0.024	-0.006	0.003	0.053	0.008	-0.050	-0.021
		(0.067)	(1.458)	(0.172)	(0.215)	(0.323)	(0.075)	(1.856)	(0.182)	(0.305)	(0.349)
**DI=0.8**	**200**	0.001*	0.042	0.001*	-0.011	-0.006	0.002	0.048	0.001*	-0.024	-0.008
		(0.047)	(0.997)	(0.123)	(0.160)	(0.227)	(0.049)	(1.238)	(0.131)	(0.215)	(0.249)
	**500**	0.001*	0.028	0.002	-0.011	0.003	0.001	0.019	0.003	-0.016	0.003
		(0.031)	(0.623)	(0.080)	(0.099)	(0.136)	(0.032)	(0.775)	(0.086)	(0.134)	(0.152)

**Table 4:** Comparison of estimation bias and standard deviation between MLE and CECF (0.001* denotes values less than 0.001)-Case C.

		Bias (MCSD)									
		MLE method					CECF method				
Case	n	*η*	μ_2_	μ_1_	$\sigma_{2}^{2}$	$\sigma_{1}^{2}$	*η*	μ_2_	μ_1_	$\sigma_{2}^{2}$	$\sigma_{1}^{2}$
**C1**	**100**	-0.038	0.136	-0.109	-0.309	-0.341	0.019	0.033	0.043	-0.280	-0.497
		(0.293)	(1.310)	(0.610)	(0.470)	(0.396)	(0.339)	(1.089)	(0.604)	(0.671)	(0.415)
**DI=0.1**	**200**	-0.014	0.092	-0.092	-0.207	-0.280	0.070	0.028	-0.001*	-0.247	-0.492
		(0.291)	(1.169)	(0.530)	(0.470)	(0.382)	(0.347)	(0.944)	(0.576)	(0.517)	(0.420)
	**500**	-0.016	0.100	-0.124	-0.121	-0.171	0.064	0.003	0.017	-0.242	-0.415
		(0.276)	(1.107)	(0.448)	(0.500)	(0.338)	(0.372)	(0.923)	(0.513)	(0.468)	(0.486)
**C2**	**100**	-0.061	0.453	-0.049	-0.197	-0.076	0.015	0.287	0.007	-0.149	-0.262
		(0.280)	(1.509)	(0.412)	(0.464)	(0.565)	(0.322)	(1.311)	(0.457)	(0.681)	(0.626)
**DI=0.3**	**200**	-0.045	0.413	-0.022	-0.068	-0.033	0.040	0.306	-0.033	-0.117	-0.230
		(0.265)	(1.230)	(0.277)	(0.438)	(0.522)	(0.305)	(0.981)	(0.349)	(0.523)	(0.585)
	**500**	-0.032	0.204	-0.008	0.004	0.003	0.032	0.186	-0.028	-0.040	-0.141
		(0.209)	(0.674)	(0.176)	(0.291)	(0.391)	(0.262)	(0.711)	(0.229)	(0.401)	(0.469)
**C3**	**100**	-0.010	0.158	0.003	-0.030	0.027	-0.001*	0.251	-0.006	-0.057	-0.013
		(0.148)	(0.459)	(0.237)	(0.292)	(0.625)	(0.167)	(1.115)	(0.233)	(0.409)	(0.606)
**DI=0.55**	**200**	-0.002	0.064	-0.011	-0.004	0.004	0.003	0.088	-0.010	-0.014	-0.005
		(0.095)	(0.458)	(0.157)	(0.198)	(0.373)	(0.116)	(0.607)	(0.167)	(0.278)	(0.429)
	**500**	-0.001*	0.022	-0.001*	-0.008	0.004	-0.003	0.033	0.001	0.004	0.018
		(0.058)	(0.253)	(0.099)	(0.122)	(0.210)	(0.062)	(0.308)	(0.103)	(0.163)	(0.253)
**C4**	**100**	-0.023	0.308	0.027	-0.100	0.055	-0.022	0.203	0.025	-0.158	0.040
		(0.079)	(0.950)	(0.183)	(0.249)	(0.400)	(0.097)	(1.179)	(0.194)	(0.343)	(0.433)
**DI=0.8**	**200**	-0.011	0.164	0.007	-0.044	0.028	-0.012	0.124	0.010	-0.078	0.020
		(0.056)	(0.648)	(0.124)	(0.179)	(0.257)	(0.067)	(0.838)	(0.135)	(0.272)	(0.294)
	**500**	-0.006	0.079	0.005	-0.026	0.022	-0.008	0.092	0.006	-0.054	0.026
		(0.034)	(0.386)	(0.080)	(0.107)	(0.148)	(0.043)	(0.557)	(0.089)	(0.179)	(0.180)

**Table 5:** Characteristics of the Five Cognitive Measures in the PREDICT-HD Study.

Cognitive Variables	Sample Sizes (n_1_, n_2_)	*μ̂*_1_	*μ̂*_2_	*σ̂*_1_	*σ̂*_2_	DI*
At 40-42 Age Window:						
SDMT	(31,231)	56.48	52.03	9.59	12.45	0.192
STROOP-C	(31,229)	85.77	78.54	9.19	14.84	0.308
STROOP-W	(31,230)	104.29	98.88	15.42	19.05	0.153
TRAILS-A	(20,153)	20.45	26.29	6.39	10.92	0.343
TRAILS-B	(20,151)	48.05	66.81	19.33	36.06	0.370
At 50-52 Age Window:						
SDMT	(65,183)	54.54	48.11	7.76	11.88	0.308
STROOP-C	(65,183)	82.11	71.90	10.96	13.02	0.336
STROOP-W	(65,183)	105.63	90.88	16.16	16.00	0.354
TRAILS-A	(42,103)	25.10	29.96	6.20	11.56	0.344
TRAILS-B	(41,104)	54.34	78.70	18.39	43.38	0.465

**Table 6:** Estimates of Model Parameters in TRAILS-B at Age Window 50-52 for the PREDICT-HD data.

Methods	*η̂*	*μ̂*_1_	*μ̂*_2_	*σ̂*_2_	*σ̂*_1_
MLE	0.26	55.87	118.05	13.83	51.14
CECF	0.31	53.96	96.95	14.15	43.37
